# Supplementary material for: Deletion of wfs1 Impairs Oligodendrocyte Precursor Cells Dorsal Distribution and Myelination Through the wfs1-hmgcs1 Axis in Zebrafish
Source: Biology (Basel). 2026 Aug 16;15(16):1402. doi: 10.3390/biology15161402 (PMC13510151; doi:10.3390/biology15161402)
Supplement: Supplementary file 1 [file biology-15-01402-s001.zip › File S1-Transcriptome sequencing data and differentially expressed genes (DEGs) in wfs1-- zebrafish at 5 dpf/1.GO/wfs1KOvsWT/GSEA/WT/SAGA_TYPE_COMPLEX/SAGA_TYPE_COMPLEX(GO_0070461).html]

Details for gene set SAGA\_TYPE\_COMPLEX(GO:0070461)[GSEA]


|  || Dataset | deg.sample |
| Phenotype | sample.cls |
| Upregulated in class | WT |
| GeneSet | SAGA\_TYPE\_COMPLEX(GO:0070461) |
| Enrichment Score (ES) | -0.44644022 |
| Normalized Enrichment Score (NES) | -1.7230077 |
| Nominal p-value | 0.013282732 |
| FDR q-value | 0.32942322 |
| FWER p-Value | 0.827 |
Table: GSEA Results Summary

  

Fig 1: Enrichment plot: SAGA\_TYPE\_COMPLEX(GO:0070461)
  
Profile of the Running ES Score & Positions of GeneSet Members on the Rank Ordered List

  

| PROBE | DESCRIPTION   (from dataset) | GENE SYMBOL | GENE\_TITLE | RANK IN GENE LIST | RANK METRIC SCORE | RUNNING ES | CORE ENRICHMENT || 1 | 503605 | supt7l |  |  | 3115 | 1.009 | 0.0163 | No |
| 2 | 568453 | supt20 |  |  | 6116 | 0.575 | -0.0128 | No |
| 3 | 557263 | trrap |  |  | 7981 | 0.425 | -0.0233 | No |
| 4 | 447818 | taf6 |  |  | 9061 | 0.363 | -0.0161 | No |
| 5 | 368510 | atxn7l3 |  |  | 12606 | 0.159 | -0.1092 | No |
| 6 | 557363 | taf6l |  |  | 13824 | 0.100 | -0.1361 | No |
| 7 | 797303 | tada2a |  |  | 15439 | 0.025 | -0.1839 | No |
| 8 | 556710 | taf10 |  |  | 17549 | -0.056 | -0.2437 | No |
| 9 | 436898 | supt3h |  |  | 18647 | -0.106 | -0.2662 | No |
| 10 | 436700 | eny2 |  |  | 19586 | -0.147 | -0.2790 | No |
| 11 | 334055 | taf5l |  |  | 20622 | -0.195 | -0.2895 | No |
| 12 | 559983 | si:dkeyp-118b1.2 |  |  | 21666 | -0.250 | -0.2940 | No |
| 13 | 321485 | taf9 |  |  | 23081 | -0.333 | -0.3007 | No |
| 14 | 402899 | tada1 |  |  | 24214 | -0.407 | -0.2902 | No |
| 15 | 554156 | tada2b |  |  | 29195 | -0.973 | -0.3364 | Yes |
| 16 | 449813 | sgf29 |  |  | 31705 | -3.724 | 0.0058 | Yes |
Table: GSEA details
[plain text format]

  

Fig 2: SAGA\_TYPE\_COMPLEX(GO:0070461)
  
Blue-Pink O' Gram in the Space of the Analyzed GeneSet

  

Fig 3: SAGA\_TYPE\_COMPLEX(GO:0070461): Random ES distribution
  
Gene set null distribution of ES for
 **SAGA\_TYPE\_COMPLEX(GO:0070461)**
